# Integrating dynamic environmental predictors and species occurrences: Toward true dynamic species distribution models

**DOI:** 10.1002/ece3.5938

**Published:** 2019-12-15

**Authors:** Pietro Milanesi, Francesca Della Rocca, Robert A. Robinson

**Affiliations:** ^1^ Swiss Ornithological Institute Sempach Switzerland; ^2^ Department of Earth and Environmental Sciences University of Pavia Pavia Italy; ^3^ British Trust for Ornithology Thetford UK

**Keywords:** climate change, dynamic predictors, land‐use change, nonstationarity, R, spatiotemporal model

## Abstract

While biological distributions are not static and change/evolve through space and time, nonstationarity of climatic and land‐use conditions is frequently neglected in species distribution models. Even recent techniques accounting for spatiotemporal variation of species occurrence basically consider the environmental predictors as static; specifically, in most studies using species distribution models, predictor values are averaged over a 50‐ or 30‐year time period. This could lead to a strong bias due to monthly/annual variation between the climatic conditions in which species' locations were recorded and those used to develop species distribution models or even a complete mismatch if locations have been recorded more recently. Moreover, the impact of land‐use change has only recently begun to be fully explored in species distribution models, but again without considering year‐specific values. Excluding dynamic climate and land‐use predictors could provide misleading estimation of species distribution. In recent years, however, open‐access spatially explicit databases that provide high‐resolution monthly and annual variation in climate (for the period 1901–2016) and land‐use (for the period 1992–2015) conditions at a global scale have become available. Combining species locations collected in a given month of a given year with the relative climatic and land‐use predictors derived from these datasets would thus lead to the development of true dynamic species distribution models (D‐SDMs), improving predictive accuracy and avoiding mismatch between species locations and predictor variables. Thus, we strongly encourage modelers to develop D‐SDMs using month‐ and year‐specific climatic data as well as year‐specific land‐use data that match the period in which species data were collected.

## Graphical abstract

In most studies using species distribution models, predictor variables are averaged over a 50‐ or 30‐year time‐period, leading to a strong bias due to monthly/annual variation between the climatic conditions in which species' locations were recorded and those used to develop species distribution models or even a complete mismatch if locations have been recorded more recently. However, thanks to recent development of open access spatially explicit databases providing monthly and annual variation of climate and land‐use conditions at global scale, it is now possible to develop dynamic species distribution models, matching dates in which species locations were collected and predictor values.

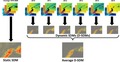

In the last decades, species distribution models (SDMs), which relate species occurrence locations with environmental predictors to estimate the probability of species occurrence to unsurveyed sites or to unsurveyed times (e.g., to predict response to climate change), experienced vigorous development at the intersection of ecology, biogeography, applied statistics, and computer science (e.g., Guillera‐Aroita, 2017; Kéry, 2011). SDMs work under the assumption that species are in equilibrium with their environment; however, it is increasingly accepted that this is unrealistic in many cases, leading to the emergence of “temporal ecology” to complement the more established study of “spatial ecology” (Ryo, Aguilar‐Trigueros, Pinek, Muller, & Rillig, 2019). This recognizes that the distributions of wild species are not static, but change/evolve through time and space (as do the environmental conditions in which they occur), in a hierarchical way, with longer‐term interannual dynamics overlaying shorter‐term intraannual dynamics. Recently developed approaches, such as spatiotemporal exploratory models (STEM; Fink et al., 2010) and dynamic occupancy models (also known as multi‐season occupancy models; Kéry & Chandler, 2012), have accounted for this spatiotemporal variation of species occurrence. Specifically, STEM is based on an ensemble or a mixture of static SDMs applied at a spatiotemporally restricted extent and then averaged over the whole extent (to account for local spatial and temporal patterns, reducing misleading extrapolation to distant regions), while dynamic occupancy models describe the occurrence at each site and the colonization and extinction probabilities from the previous time step.

However, even in these recent techniques, as well as in the more traditional static SDMs, the environmental predictors considered to estimate species occurrence are basically static. For instance, most of the studies applying SDMs attempt to predict species' distribution under future climate change scenarios (Titeux et al., 2016), but through static climatic predictors; specifically, most of these studies use climatic data that are averaged over a 50‐ (1950–2000, Hijmans, Cameron, Parra, Jones, & Jarvis, 2005) or 30‐year period (1970–2000; Fick & Hijmans, 2017). Thus, there could be a strong bias due to monthly/annual variation between the climatic conditions in which mobile species' locations were recorded and those used to develop SDMs, or even a complete mismatch in case of species locations collected after the year 2000. This problem becomes more acute with the increasing prevalence of climatic extremes (e.g., the summer of 2003 in Europe; Jentsch & Beierkuhnlein, 2008), either models using averaged baseline data are increasingly unrepresentative, or, if such events are included in the baseline, the mean values may be unduly skewed (or at least the variability about them, which is often unaccounted for, is increased).

Currently, there is wide consensus that both climate and land‐use change are among the most important threats to biodiversity and ecosystem services worldwide (IPBES, 2018, 2019; Maxwell, Fuller, Brooks, & Watson, 2016; Scheffers et al., 2016), and often interact (Oliver & Morecroft, 2014). However, the impact of land‐use change has rarely been explored in SDMs to date (Titeux et al., 2016) and the few studies (Milanesi, Breiner, Puopolo, & Holderegger, 2017; Newbold et al., 2015; Radinger et al., 2016) that have considered it have used static predictors (e.g., neglecting the effect of the destruction and modification of natural habitats). Actually, while the use of climate change projections is a commonplace, land‐use change projections have more rarely been used in species forecasting (Bateman et al., 2013). This may be because they are not as widely available as climatic predictions are; incorporating these will be of critical importance since the interactions among multiple drivers of global change have recently been identified as a major cause of uncertainty in climate change attribution projection (Oliver & Morecroft, 2014; Parmesan et al., 2013), in part because multiple environmental pressures may have a greater joint impact than when operating in isolation (Ostberg, Schaphoff, Lucht, & Gerten, 2015). Thus, in the absence of integrative multi‐driver approaches, limited understanding of how interactions among drivers affect species distribution will be likely to hamper reliable projections (Titeux et al., 2016).

Although in some cases the distribution and/or diversity of species in different seasons may be better explained by a single predictor rather than multiple seasonally specific ones (e.g., Lennon, Greenwood, & Turner, 2000), SDMs that do not account for nonstationarity of climatic and land‐use predictors may suffer from at least three problems: (a) The probability of species' occurrence at particular locations could be inaccurate due to the (temporally) averaged values in the predictors; (b) estimated slopes of predictor relationships could be biased; (c) some predictors may be wrongly identified as determinants of species' occurrence and/or may mask the real determinants, resulting, for example, in misleading inference or the wrong identification of areas of conservation importance. For example, species distribution may be negatively related to summer temperatures, but positively related to those in winter (Kawamura, Yamaura, Senzaki, Ueta, & Nakamura, 2019), patterns that would be masked by simply using annual values.

Treating the environment as static has, in part, been enforced by a lack of relevant data; however, researchers have recently developed open‐access spatially explicit databases providing monthly and annual variation of climate and land‐use conditions at global scale. For example, Karger et al. (2018) produced CHELSAcruts, a monthly climate (e.g., precipitation, maximum, and minimum temperature) global dataset at ~1 km spatial resolution for the period 1901–2016 (http://chelsa-climate.org/chelsacruts/), while Abatzoglou, Dobrowski, Parks, and Hegewisch (2018) produced TerraClimate, a monthly climate (e.g., precipitation, temperature, and wind speed) and climatic water balance (e.g., actual and potential evapotranspiration, soil moisture) global dataset at ~4 km spatial resolution for the period 1958–2015 (currently updated to the year 2017; https://climate.northwestknowledge.net/TERRACLIMATE/index_directDownloads.php). On the other side, the European Space Agency (ESA – European Space Agency, 2017) have recently developed annual global land cover time series from 1992 to 2015 (at ~0.3 km resolution), including two levels of details and a total of 37 land‐use category (e.g., different types of croplands, forests, shrublands, and grasslands as well as urban areas, water bodies, and glaciers, etc.; https://www.esa-landcover-cci.org/?q=node/175).

These datasets have the potential to provide important inputs for ecological studies requiring high spatial resolution and time‐varying climate and land‐use data, at both local and global scales. Specifically, considering occurrences of a species (sensitive to both climate and land‐use change), collected during the period 1992–2015, it is currently possible to model its distribution relating species' locations collected in a given month of a given year, matching climatic and land‐use conditions. These dynamic species distribution models (D‐SDMs) would be especially useful to model the occurrence of species displaying different patterns of distribution through seasons (e.g., migratory birds, insects, plants, etc.) and/or years (e.g., any species undergoing changes in its distribution).

To demonstrate how predicted patterns of species occurrence could vary between static and dynamic distribution models, we simulated a set of species recording locations (*n* = 3,000) in central Europe (5°–11° E; 45°–48° N) such as might be obtained from a citizen science survey. We used the ensemble predictions of four algorithms, namely boosted regression trees (BRT; Friedman, 2001), generalized additive models (GAM; Hastie & Tibshirani, 1990), generalized linear models (GLM; McCullagh & Nelder, 1989), and random forests (RF; Breiman, 2001) in R (R Development Core Team, 2013) although D‐SDMs could be developed using any appropriate algorithm. Similarly, while one can use any of the widely used validation statistics (Lecocq, Harpke, Rasmont, & Schweiger, 2019), to compare the predictive accuracy of both static and D‐SDMs, in our simulations we considered the area under the receiver operating characteristic curve (AUC) and the true skills statistic (TSS). AUC ranges between 0 and 1 (worse than a random model and best discriminating model, respectively) while TSS varies between −1 and 1 (higher values indicate a good predictive accuracy, while 0 indicates random prediction). By using a random subsample of 90% of the locations to calibrate the models and the remaining 10% to evaluate them (Thuiller, Lafourcade, Engler, & Araújo, 2009), we carried out 10‐fold cross‐validations to test the predictive accuracy of both static and D‐SDMs (see average values below).

In this example, pooling virtual species locations (e.g., collected in the period 2010–2015) and ignoring the temporal mismatch between them and the predictor variables (from the WorldClim 2 database; Fick & Hijmans, 2017) leads to biased estimation of species distribution (overestimated in this case; AUC = 0.893; TSS = 0.809). On the other side, we developed D‐SDMs splitting virtual species locations and accounting for year‐specific predictors (from the CHELSAcruts database; Karger et al., 2018) which showed, together with interannual fluctuations, a relatively limited average species distribution (Figure 1; AUC = 0.968; TSS = 0.898). One of the main drawbacks of this approach is that for each time period (i.e., year in our example) the number of species locations could be much lower than those of static SDMs (which pool data) leading, potentially, to a higher influence of unusual observations (which may be diluted by pooling) and possible model overfitting (i.e., model fits the calibration data too closely, in environmental space, but fails to predict independent evaluation data accurately; Radosavljevic & Anderson, 2014). To overcome this, we pooled virtual species locations but associated each location to its relative year‐specific predictor values and thus developed a single SDM to predict virtual species distribution under different year‐specific conditions, which again showed a more restricted average species distribution of D‐SDMs compared with the static SDMs (Figure 2; AUC = 0.943; TSS = 0.871). As well as reducing the likelihood overfitting, this approach also has the benefit of accounting for interannual variation (e.g., species adaptability to climatic variations) in the prediction of species distribution under different year‐specific conditions and thus also improves estimation during climatic anomalies (which have become larger and more frequent in the last decade due to climate change; Ummenhofer & Meehl, 2017). The recently developed “ensemble of small models” approach (Breiner, Guisan, Bergamini, Nobis, & Anderson, 2015) provides an alternative way to reduce overfitting.

**Figure 1:**
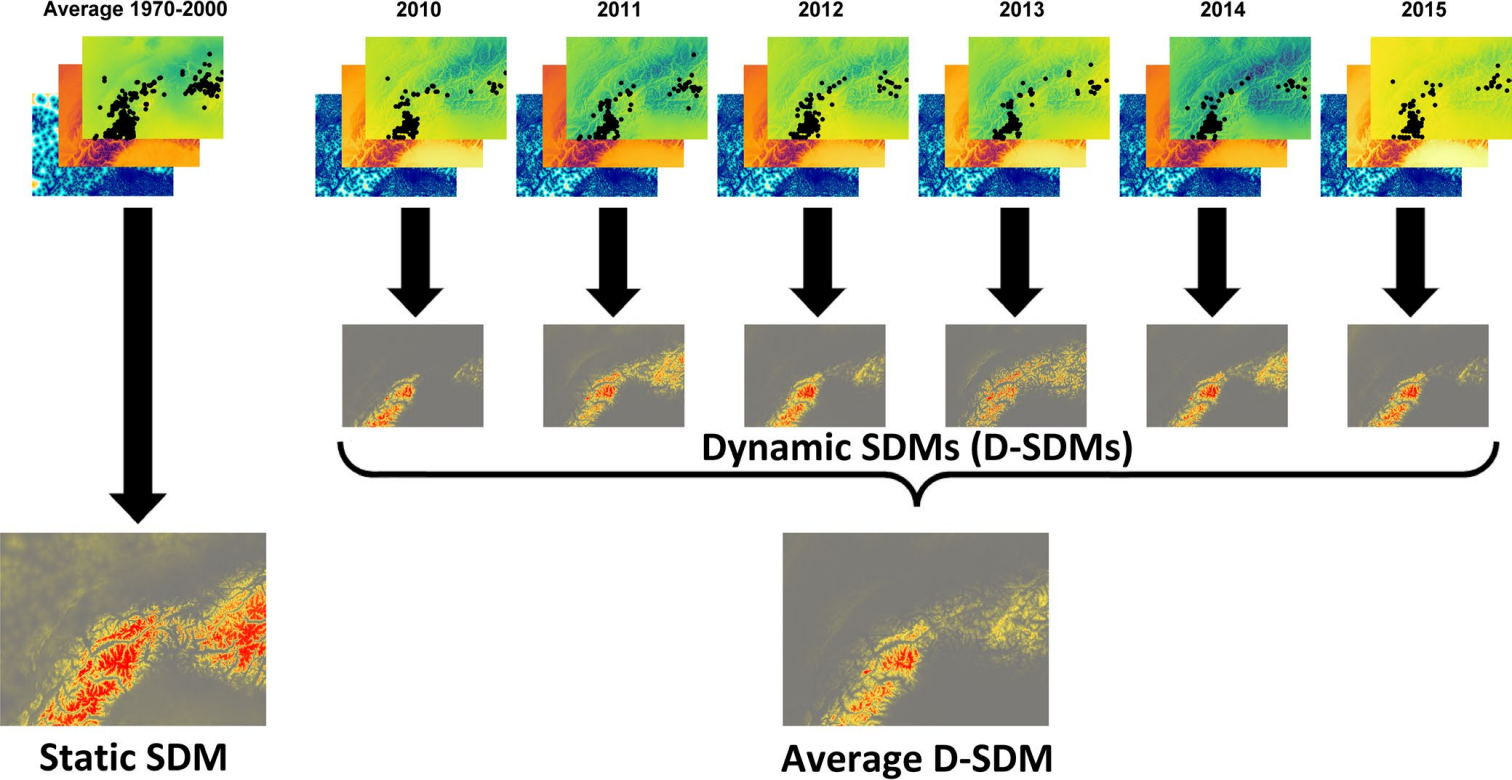
Differences between static and dynamic species distribution models. Predictor variables (in this case Bio 3—Isothermality, Bio 10—Mean temperature of the warmest quarter, and Bio 17—Precipitation of the driest quarter) came from WorldClim 2 database (Average 1970–2000; left) and CHELSAcruts (annually for 2010–2015, right), together with relative virtual species locations (black dots) collected in the period 2010–2015, are shown on the first line. Resulting maps of dynamic (annual) species distribution models are shown on the second line while those of static and averaged dynamic species distribution models are shown on the third line. Red‐gray scale indicates high–low probability of occurrence

**Figure 2:**
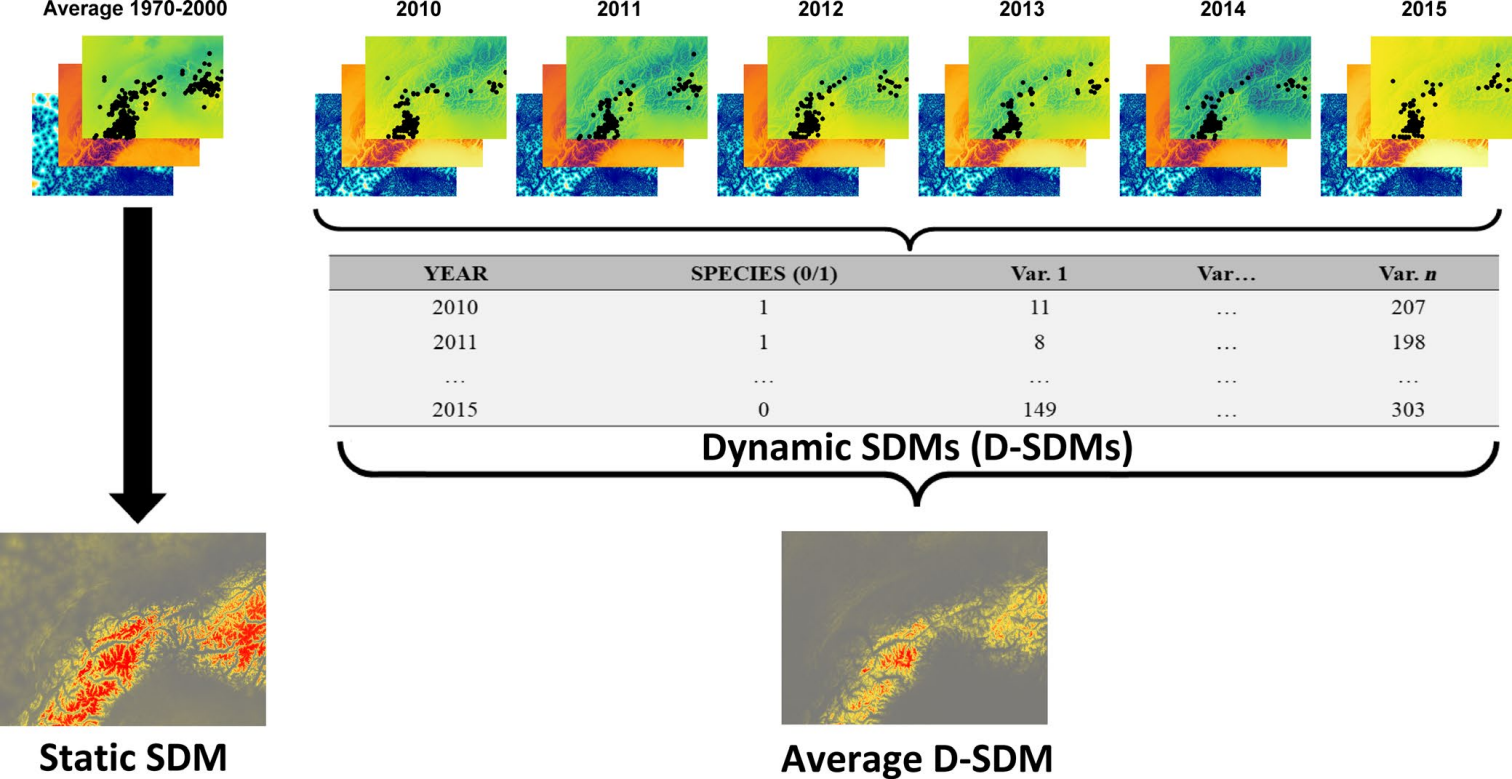
Differences between static and averaged dynamic species distribution models. Predictor variables and static model as in Figure 1, for the dynamic model year‐specific predictor values are pooled for 2010–2015 into a single database, shown on the second line. Resulting map of static and averaged dynamic species distribution models is shown on the third line. Red‐gray scale indicates high–low probability of occurrence

Thus, D‐SDMs can show higher predictive accuracy compared with static SDMs and could also improve the outcomes of STEM and dynamic occupancy models, providing more robust trends of species occurrence. For these reasons, we strongly encourage modelers to develop D‐SDMs in order to provide more accurate estimates of species distribution. Such approaches are likely to be particularly valuable in more climatically variable regions and habitats (e.g., Reside, Wal, Kutt, & Perkins, 2010) and may become more important as climatic variability is predicted to increase (Kharin, Zwiers, Zhang, & Hegerl, 2007). However, species data quality (i.e., availability of information about the year and month in which species locations were collected), as well as the lack of open‐access databases for nonterrestrial environments (e.g., pH concentration for freshwater biotopes), can strongly limit the application of D‐SDMs and thus further improvements of both species data quality and accessibility of additional spatiotemporal environmental data are needed (e.g., Wetzel et al., 2018).

Finally, we urge researchers to make use of newly available datasets coming online to include both climate and land‐use dynamic predictors, as the strength of impacts on biodiversity will likely depend on the interaction between these (Oliver & Morecroft, 2014). This will ensure, perhaps in combination with recently developed methods for generating high‐resolution climate projections (Maclean, 2019), that future decision‐making, such as prioritizing areas for conservation (e.g., Jones, Watson, Possingham, & Klein, 2016), more robustly anticipates the response of biodiversity to future climate and land‐use changes.

## CONFLICT OF INTEREST

None declared.

## AUTHOR CONTRIBUTIONS

PM conceived and designed the overall study, PM and RAR wrote the manuscript, FDR and PM conducted the statistical analyses and designed the figures. FDR prepared the figures. All three authors contributed substantially to revisions of the paper.

## Data Availability

Climatic GIS layers used for analysis in this paper are freely available at http://chelsa-climate.org/chelsacruts/ and http://worldclim.org/version2.
